# Dysregulation of a Subset of Circulating and Vesicle-Associated miRNA in Pancreatic Cancer

**DOI:** 10.3390/ncrna10030029

**Published:** 2024-05-01

**Authors:** Giulia Girolimetti, Iulia Andreea Pelisenco, Leonardo Henry Eusebi, Claudio Ricci, Beatrice Cavina, Ivana Kurelac, Tiziano Verri, Matteo Calcagnile, Pietro Alifano, Alessandro Salvi, Cecilia Bucci, Flora Guerra

**Affiliations:** 1 Department of Biological and Environmental Sciences and Technologies (DiSTeBA), University of Salento, Via Provinciale Lecce-Monteroni 165, 73100 Lecce, Italy; giulia.girolimetti@unisalento.it (G.G.); tiziano.verri@unisalento.it (T.V.); matteo.calcagnile@unisalento.it (M.C.); flora.guerra@unisalento.it (F.G.); 2 Department of Molecular and Translational Medicine, University of Brescia, Viale Europa 11, 25123 Brescia, Italy; i.pelisenco@unibs.it (I.A.P.); alessandro.salvi@unibs.it (A.S.); 3 Department of Medical and Surgical Sciences (DIMEC), University of Bologna, Via Massarenti 9, 40138 Bologna, Italy; leonardo.eusebi@unibo.it (L.H.E.); claudio.ricci6@unibo.it (C.R.); beatrice.cavina2@unibo.it (B.C.); ivana.kurelac@unibo.it (I.K.); 4 Gastroenterology Unit, IRCCS Azienda Ospedaliero-Universitaria Di Bologna, Via Massarenti 9, 40138 Bologna, Italy; 5 Pancreatic Surgery Unit, IRCCS Azienda Ospedaliero-Universitaria Di Bologna, Via Massarenti 9, 40138 Bologna, Italy; 6 Centre for Applied Biomedical Research (CRBA), University of Bologna, Via Massarenti 9, 40138 Bologna, Italy; 7 Department of Experimental Medicine (DiMeS), University of Salento, Via Provinciale Lecce-Monteroni 165, 73100 Lecce, Italy; pietro.alifano@unisalento.it

**Keywords:** miRNA, extracellular vesicles, pancreatic cancer, circulating miRNA, molecular biomarkers, chemoresistance

## Abstract

Pancreatic ductal adenocarcinoma (PDAC) is one of the most aggressive neoplasia, characterized by early metastasis, low diagnostic rates at early stages, resistance to drugs, and poor prognosis. There is an urgent need to better characterize this disease in order to identify efficient diagnostic/prognostic biomarkers. Since microRNAs (miRNAs) contribute to oncogenesis and metastasis formation in PDAC, they are considered potential candidates for fulfilling this task. In this work, the levels of two miRNA subsets (involved in chemoresistance or with oncogenic/tumor suppressing functions) were investigated in a panel of PDAC cell lines and liquid biopsies of a small cohort of patients. We used RT-qPCR and droplet digital PCR (ddPCR) to measure the amounts of cellular- and vesicle-associated, and circulating miRNAs. We found that both PDAC cell lines, also after gemcitabine treatment, and patients showed low amounts of cellular-and vesicle-associated miR-155-5p, compared to controls. Interestingly, we did not find any differences when we analyzed circulating miR-155-5p. Furthermore, vesicle-related miR-27a-3p increased in cancer patients compared to the controls, while circulating let-7a-5p, miR-221-3p, miR-23b-3p and miR-193a-3p presented as dysregulated in patients compared to healthy individuals. Our results highlight the potential clinical significance of these analyzed miRNAs as non-invasive diagnostic molecular tools to characterize PDAC.

## 1. Introduction

Pancreatic ductal adenocarcinoma (PDAC) is an aggressive malignancy, representing one of the leading causes of cancer-related death with a 5-year relative survival rate of 12% and a progressive annual increase in the number of diagnoses [1]. Early metastasis, high local recurrence rate, and chemoresistance represent the most important factors driving lethality [2]. Surgical resection is an approach associated with a better prognosis but only 10–20% of patients are eligible [3,4]. Indeed, localized pancreatic cancer is usually asymptomatic or occurs with ill-defined symptoms, and diagnostic biomarkers for earlier stage disease or efficient screening methods are still lacking [2]. Therefore, most patients are diagnosed with advanced stage cancer and distant metastasis when only therapeutic options are available [3,5]. Chemotherapy for metastatic PDAC consists of gemcitabine alone or combined with nab-paclitaxel/erlotinib, otherwise FOLFIRINOX (5-FU, leucovorin, irinotecan, and oxaliplatin) is employed, a most recent approach with increased toxicity [6,7,8]. The benefit of chemotherapy is frequently minimal because PDAC is characterized by a high refractoriness to pharmacological treatments. This is due, in part, to the activity of ATP-binding cassette (ABC) proteins that actively mediate the efflux of drugs from cancer cells across the plasma. The upregulation of these pumps may cause the reduction in chemotherapeutic agent concentration in cancer cells, limiting their efficiency [9]. Among ABC proteins, multidrug resistance protein 1 (MDR1), also known as P-glycoprotein or P-gp (gene symbol *ABCB1*), was the first to be related to chemoresistance, contributing to the development of multidrug resistance (MDR) in different types of cancer [10]. An MDR1 pump can transport among a large variety of anticancer drugs, including gemcitabine, and is associated with PDAC chemoresistance [11,12]. Furthermore, other genes surrounding the ABCB1 locus, referred to as *ABCB1* amplicon, were reported to contribute to the establishment of the MDR phenotype and were found to be overexpressed in MDR tumors [13].

Non-coding RNAs (ncRNAs), such as microRNA (miRNA), are usually dysregulated in cancer cells, including PDAC. In tumorigenesis and cancer progression, differential expression of miRNAs may reflect the role of the gene targets: the downregulation of tumor-suppressor miRNAs increases the activity in oncogenes, while the upregulation of oncogenic miRNAs (onco-miRNAs) decreases the activity in tumor suppressor genes [14,15]. Furthermore, they are widely involved in the development of cancer drug resistance by modulating the expression of specific target genes involved in cellular processes such as apoptosis, autophagy, epithelial-to-mesenchymal transition and drug efflux [16,17,18,19,20,21].

Due to the different expressions in neoplastic compared to non-pathological conditions and between different phases of tumor progression, miRNAs are currently evaluated as possible biomarkers for cancer diagnostic, prognostic, and therapeutic choices [22]. MicroRNAs have been reported as free molecules in circulation but may also be a part of the cargo carried by extracellular vesicles (EVs) where they are usually more stable [23,24]. EVs are membranous structures with a diameter of 50–1000 nm containing lipids, proteins, metabolites, and nucleic acids such as DNA, mRNAs, and miRNAs [25]. Proteins and nucleic acids such as miRNAs from small EVs (sEV < 200 nm) were reported to play pivotal roles in tumorigenesis, chemoresistance, and metastases formation [26]. The sEVs are present in all body fluids, such as blood, saliva, urine, pancreatic duct fluid, ascites and cerebrospinal fluid [27,28,29,30,31,32,33], which transport these vesicles and their information to distal tissues or organs [34]. Furthermore, sEVs are highly stable in biological fluids and their abundance permits isolation from small volumes of serum or plasma. Taken together, these characteristics make sEVs good biomarker candidates for disease diagnosis, prognosis, and surveillance.

Developing novel and efficient diagnostic, prognostic, and therapeutic markers for pancreatic cancer is a great challenge and there is an immediate need to increase the survival rates of patients. In PDAC, sEVs are involved in carcinogenesis, progression, invasion and dissemination, immunosuppression, and chemoresistance, and the analysis of their cargo could be used for diagnosis and prognosis. In this context, both free circulating and sEV-associated miRNAs were described as PDAC biomarker candidates [25,35]. Here, we tested the expression of a set of miRNAs related to chemoresistance, tumorigenesis, and progression in PDAC cell lines and patients to select vesicle-associated and circulating biomarkers candidates that may be clinically useful for the molecular characterization of PDAC patients. 

## 2. Results

### 2.1. A Novel Set of miRNAs Is Predicted to Represent Molecular Biomarkers Candidates in PDAC 

**Figure 1:**
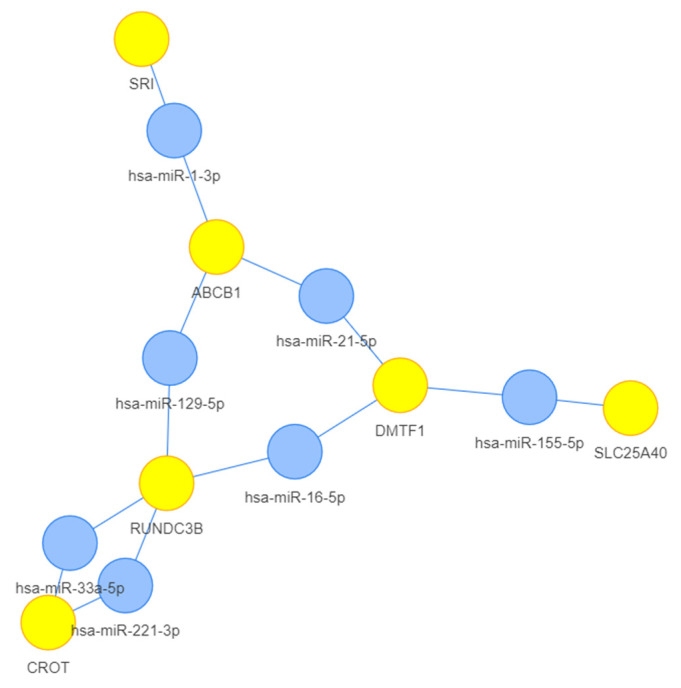
*ABCB1* amplicon-associated miRNAs based on MIENTURNET (http://userver.bio.uniroma1.it/apps/mienturnet/)/ (accessed on 14 March 2024) analysis [MicroRNA/Genes Network: miRTarBase; default parameters, i.e., threshold for the minimum number of miRNA-target interactions: 2; threshold for the adjusted *p*-value (FDR): 1. Filter by Evidence categories: Strong and Weak]. The 6 genes (yellow) and 7 miRNAs (light blue) of the network are represented. *p*-values and FDR (False Discovery Rate) are reported in Table S1.

To select a set of miRNAs that could be dysregulated in PDAC and represent possible candidate molecular biomarkers, we decided to focus on one of the most important actors in cancer chemoresistance—MDR1—to identify the miRNAs targeting *ABCB1* amplicon. An in silico approach was applied and the following elements, contained in the *ABCB1* amplicon [13], were submitted for bioinformatic analysis: *DMTF1*, *TMEM243*, *TP53TG1*, *CROT*, *ABCB4*, *HNRNPA1P9*, *ABCB1*, *RUNDC3B*, *SLC25A40*, *DBF4*, *ADAM22*, *LOC105375386*, *SRI*, *LOC102723885* (aka *SRI-AS1*). A set of miRNAs was selected using MIENTURNET (MicroRNA ENrichment TURned NETwork) http://userver.bio.uniroma1.it/apps/mienturnet/ (accessed on 14 March 2024) [36,37,38,39] which included hsa-miR-1-3p, hsa-miR-129-5p, hsa-miR-21-5p, hsa-miR-155-5p, hsa-16-5p, hsa-miR-33a-5p and hsa-miR-221-3p, comprising the network of the genes *DMTF1*, *CROT*, *ABCB1*, *RUNDC3B*, *SLC25A40*, and *SRI* (Figure 1, Table S1). Notably, among them, hsa-miR-21-5p, hsa-miR-155-5p and hsa-miR-221-3p were already been reported in a reference list of miRNAs pre-selected by our group [25]. Identical network results were also obtained using miRTargetLink 2.0 https://ccb-compute.cs.uni-saarland.de/mirtargetlink2/ (accessed on 14 March 2024) [40] analysis (Table S2). Based on the FDR values in Table S1, to select the most relevant miRNAs for the analysis, miRNAs with a FDR value higher then 0,202 were not taken into consideration. miR-16a-5p showed a higher *p*-value and FDR (0,237 and 0,308, respectively, Table S1) making it a poor candidate, and thus it was excluded from the analysis. In addition, based on the current literature on the EVs content and their role in PDAC, we decided to take into consideration an additional 3 miRNAs—hsa-let-7a-5p, hsa-miR-301a-3p and hsa-miR-27a-3p—due to their prominent association with EVs and reported role in PDAC pathogenesis [25]. Taken together, a set of 9 miRNAs were selected. 

### 2.2. Pancreatic Cancer and Non-Cancer Cell Lines Exhibit Different Levels of Cellular and Vesicle-Associated miRNAs

Numerous studies have indicated that abnormal miRNAs expression plays an important role in the tumorigenesis, progression, and chemoresistance of PDAC, and may be used as biomarkers in the identification of cancer diagnosis. Thus, we tested the selected set of miRNAs in a panel of PDAC cell lines with different chemo-sensitivities, genotypic, and phenotypic characteristics (BxPC-3, YAPC, PANC-1, MIA PaCa-2) compared to pancreatic duct epithelial cells H6c7 to determine the differential levels of cellular miRNAs and sEV-miRNAs. The relative quantification was measured with Real Time PCR (RT-qPCR) between cancer and non-cancer cell lines. It was not possible to detect miR-1-3p in H6c7, BxPC-3, and MIA PaCa-2 cells and in H6c7, BxPC-3, YAPC, and MIA PaCa-2 cell-derived sEVs. Moreover, in YAPC and PANC-1 cells, and in PANC-1 sEVs, the miR-1-3p amount was detected at cycle thresholds close to the established cut-off value. Therefore, the latter result was considered unreliable, and was excluded from the analysis. Apart from miR-1-3p, all the other miRNAs were detected in both cells and cell-derived sEVs, suggesting that they may be considered as part of the sEVs cargo.

**Figure 2:**
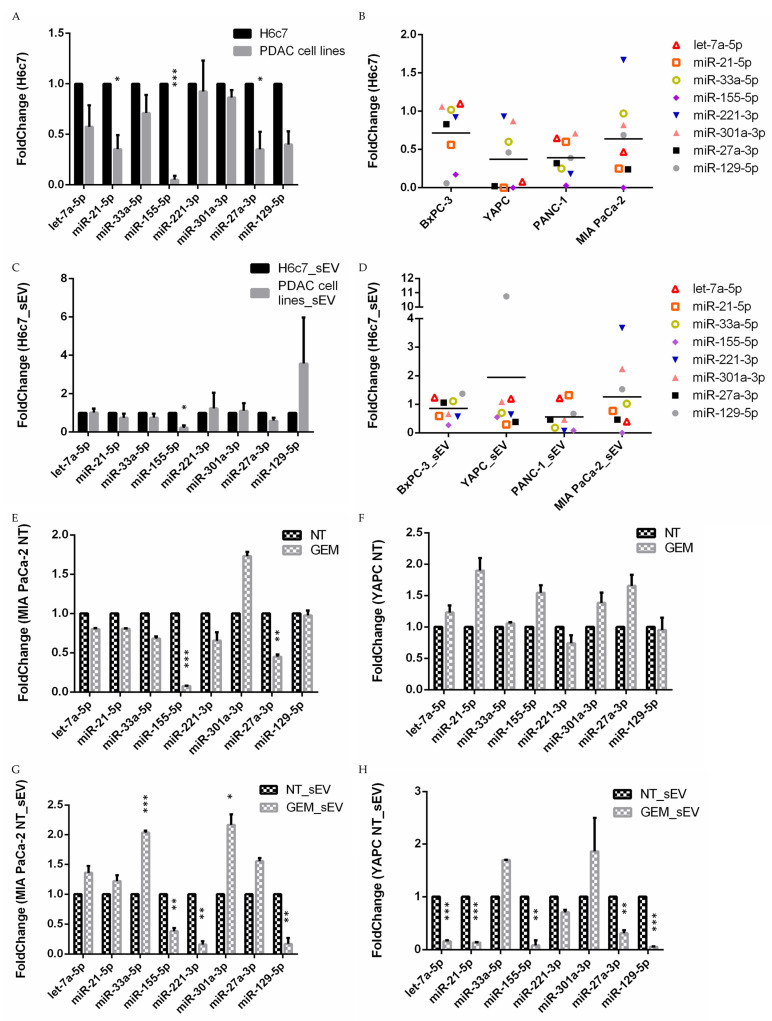
MicroRNA relative expression in the PDAC cell lines and in sEVs evaluated by RT-qPCR. (**A**) Intracellular expression of the selected miRNAs in PDAC cell lines compared to the control H6c7 cells was shown. The mean of BxPC-3, YAPC, PANC-1, and MIA PaCa-2 is reported (PDAC cell lines). (**B**) miRNA levels in different pancreatic cancer cell lines. Fold-change between each PDAC cell line compared to the control H6c7 cells was reported, the grand mean is shown. (**C**) Levels of the selected sEV-miRNA in the medium of PDAC cell lines compared to the control H6c7 cells. The mean of BxPC-3, YAPC, PANC-1, and MIA PaCa-2 is reported (PDAC cell lines). (**D**) miRNA levels in the sEVs from different pancreatic cancer cells. Fold-change between each PDAC cell line compared to the control H6c7 cells was reported, the grand mean is shown. (**E**,**F**) Intracellular expression of the selected miRNAs in MIA PaCa-2 and YAPC cells after 72 h treatment with 20 nM gemcitabine (GEM) compared to the untreated cells (NT). (**G**,**H**) Levels of the selected sEV-miRNAs purified from MIA PaCa-2 and YAPC cells after 72 h treatment with 20nM gemcitabine (GEM_sEV) compared to untreated cells (NT_sEV). All reported data are mean  +  SEM (* *p* < 0.05; ** *p* < 0.01; *** *p* < 0.001).

Considering the expression of cellular miRNAs (Figure 2A,B and Figure S1A), it is possible to note that the levels of miR-21-5p, miR-27a-3p, and especially of miR-155-5p, were significantly lower in PDAC cell lines compared to the normal H6c7. Interestingly, miR-155-5p was less abundant also in sEVs from PDAC cells compared to the sEVs from control H6c7 cells (Figure 2C,D and Figure S1B). Taken together, miR-155-5p showed the same trend as cellular- and vesicle-associated miRNA, highlighting the different levels in pancreatic cancer and non-cancer cell lines. Furthermore, considering the grand mean for the analyzed miRNAs, it was possible to note that the PDAC cell lines displayed a general reduction in mean cellular expression compared to the control cell line H6c7 (Figure 2B). This is particularly evident for the YAPC and PANC-1 cell lines. In particular, YAPC derived from ascitic fluid, representing aggressive and advanced stage cancer cells, shows the lowest levels of hsa-miR-155-5p, hsa-miR-27a-3p, hsa-miR-21-5p, and hsa-let-7a-5p compared to the other cancer cell lines (Figure 2B). When sEVs are considered, PANC-1 exhibits the lowest grand mean and YAPC sEVs show high levels of miR-129-5p, while miR-221-3p and miR-301a-3p levels are elevated in MIA PaCa-2 sEVs compared to the H6c7 sEVs (Figure 2D).

#### Gemcitabine Treatment Alters miRNA Profile in Sensitive and Resistant Pancreatic Cancer Cell Lines

With the aim of investigating the role of the selected miRNA in pancreatic cancer chemoresistance, the cell viability of the different PDAC cell lines was evaluated in the presence of gemcitabine (Figure S1C). Cells were treated for 72 h with increasing concentrations of the drug. The results showed that MIA PaCa-2 cells exhibited the higher sensitivity to gemcitabine, while YAPC cells appeared to be poorly affected by the same concentrations of the drug. To identify possible miRNA candidates as potential molecular biomarkers for gemcitabine chemotherapy response, we considered the miRNA levels in MIA PaCa-2 and YAPC cells. MIA PaCa-2, the most chemosensitive cell line, showed a higher levels of miR-221-3p both in cells and sEVs, and high levels of miR-301a-3p in sEVs (Figure 2B,D) while the YAPC cell line, in addition to exhibiting the lowest levels of miR-27a-3p, miR-21-5p, let-7a-5p, and miR-155-5p intracellularly (Figure 2B), had a higher amount of miR-129-5p in sEVs (Figure 2D) compared to the H6c7 control cell line.

Furthermore, to better understand the relation of these miRNAs to chemoresistance, PDAC cell lines were treated with gemcitabine for 72 h (20 nM, which is the EC50, the half-maximal effective concentration, of the more sensitive MIA PaCa-2 cells) and the intracellular and vesicular levels of miRNAs of treated cells were compared to non-treated cells (Figure 2E–H and Figure S1D–G). Interestingly, after gemcitabine treatment, MIA PaCa-2 cells exhibited decreased levels of intracellular miR-155-5p and miR-27a-3p (Figure 2E). In contrast, in the most gemcitabine-resistant YAPC cell line, no significant differences were found between the intracellular miRNA expression of treated and untreated cells (Figure 2F). Analysis of the same miRNAs in the vesicular fraction of MIA PaCa-2 cells revealed a significant decrease in miR-155-5p, miR-221-3p, and miR-129-5p and a higher amount of miR-33a-5p and miR-301a-3p (Figure 2G), while all miRNAs in the sEVs of YAPC were downregulated, except for miR-33a-5p, miR-301a-3p, and miR-221-3p (Figure 2H). Taking these data together, the differences detected between the MIA PaCa-2 and YAPC cell lines may be useful in identifying miRNAs that could be related to sensitivity or resistance to gemcitabine treatment. Indeed, our results showed that gemcitabine treatment in sensitive MIA PaCa-2 cells caused a reduction in intracellular miR-155-5p and miR-27a-3p of the vesicle-associated miR-221-3p and an increase in vesicle-associated miR-33a-5p and miR-301-3p levels that are not present in the YAPC cell line. On the other hand, in the YAPC cell line, although gemcitabine treatment causes no significant differences in intracellular miRNA levels, low levels of let-7a-5p, miR-21-5p, and miR-27a-3p in the vesicular component may be related to a gemcitabine-resistant phenotype.

### 2.3. Peripheral Blood-Derived Circulating and Vesicle-Associated miRNAs Are Present at Different Levels in Pancreatic Cancer Patients and Control Individuals

To understand whether the results achieved in PDAC cell lines may be translated in patients, in terms of identifying potential liquid biopsy molecular biomarker candidates among the tested set of miRNAs, the levels of sEV-associated and circulating miRNAs were measured in serum or plasma in a small cohort of patients diagnosed with pancreatic cancer (EXOPANC01-11 for vesicle-associated miRNAs and EXOPANC03-11 for circulating miRNAs) (Table 1) and compared to healthy donors (Table S3). Samples were collected at the time of diagnosis, before chemotherapy or cytoreduction in the tumor. sEV and circulating RNA, including microRNA, were purified from serum and plasma and the relative quantification was performed with RT-qPCR. It was not possible to take into consideration some of the selected miRNAs, miR-1-3p and miR-129-5p in particular, as these were either undetected or found expressed at cycle thresholds close to the established cutoff value, suggesting that these miRNAs are not PDAC-related biomarkers. All the other miRNAs were detected both in peripheral blood-derived sEVs cargo and as freely circulating molecules, in both patients and controls.

**Figure 3:**
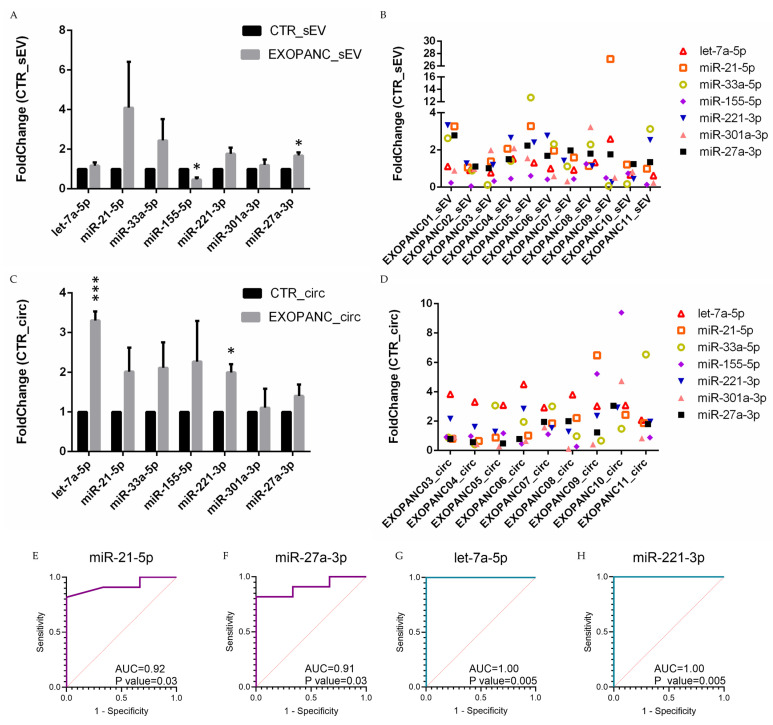
Relative quantification of small EV-associated and circulating miRNAs, evaluated by RT-qPCR in EXOPANC patients compared to the control individuals. (**A**) Levels of selected vesicle-associated miRNAs in pancreatic cancer patients EXOPANC01-11 (EXOPANC_sEV) compared to their relative controls (CTR_sEV). Data are mean  +  SEM (* *p* < 0.05). (**B**) sEV-associated miRNA levels in single patients (EXOPANC01-11). Fold-change between each EXOPANC patient compared to the controls was reported. (**C**) Levels of selected circulating miRNAs in pancreatic cancer patients EXOPANC03-11 (EXOPANC_sEV) compared to their relative controls (CTR_sEV). Data are mean  +  SEM (* *p* < 0.05; *** *p* < 0.001); (**D**) Circulating miRNA levels in single patients (EXOPANC03-11). Fold-change between each EXOPANC patient compared to controls was reported; (**E**,**F**) ROC curves of sEV-associated miRNAs detected in EXOPANC patients (n = 11) and controls (n = 3). Violet ROC curves indicates a significant AUC: (**E**) miR-21-5p (0.92, with 95% confidence interval 0.77–1.00) and (**F**) miR-27a-3p (0.91, with 95% confidence interval 0.75–1.00). (**G**,**H**) ROC curves of circulating miRNAs detected in EXOPANC patients (n = 9) and the controls (n = 4). Green ROC curves indicate a significant AUC: (**G**) let-7a-5p and (**H**) miR-221-3p (1.00). Sensitivity and 1-Specificity are represented for all the obtained deltaCt values. Red dotted line represents AUC threshold of 0.50. AUC: area under the curve, ROC: Receiver operating characteristic.

Regarding the analysis of sEV-associated miRNAs, taking into consideration the differences between pancreatic cancer patients (EXOPANC) and healthy donors, it is possible to note that the reduction in miR-155-5p measured in cancer cell lines and their sEVs compared to the normal H6c7 cells was confirmed in peripheral blood-derived sEVs of pancreatic cancer patients compared to the controls (Figure 3A,B and Figure S2A). This reduction was not observed for circulating miR-155-5p, whose levels were comparable between patients and the controls. On the other hand, we found high levels of sEV-miR-27a-3p (Figure 3A,B and Figure S2A), circulating let-7a-5p and miR-221-3p (Figure 3C,D and Figure S2B) in EXOPANC patients compared to healthy individuals. Overall, the levels of vesicle-associated and circulating miRNAs indicated a tendency to increase in pancreatic cancer patients compared to the controls, in particular, sEV-miR-21-5p, which exhibited the higher fold-change (Figure 3A). Moreover, it was possible to note that sEVs-miR-21-5p showed higher levels in patients diagnosed with stage IV and distant metastasis such as EXOPANC01, 04, 05, and mostly in EXOPANC09, suggesting that the level of this miRNA may increase in the advanced stages of disease (Figure 3B and Figure S2A), but a larger cohort is needed to investigate this relation.

Next, to define the biomarker sensitivity and specificity of the candidate miRNAs, we performed receiver operating characteristic analysis (ROC) (Figure 3E–H and Figure S2C,D). Despite the low number of cases and controls, significant results were observed for sEV-derived miR-21-5p and miR-27a-3p (Figure 3E,F), as well as for circulating let-7a-5p and miR-221-3p (Figure 3G,H). 

Taken together, these results suggest that high levels of sEV-miR-27a-3p, circulating let-7a-5p, and circulating miR-221-3p should be considered as possible molecular biomarkers for PDAC to include in a future prospective study in a larger cohort.

### 2.4. Circulating Levels of Selected miRNAs with Oncogenic or Tumor-Suppressing Functions in PDAC Patients and in Healthy Individuals

**Figure 4:**
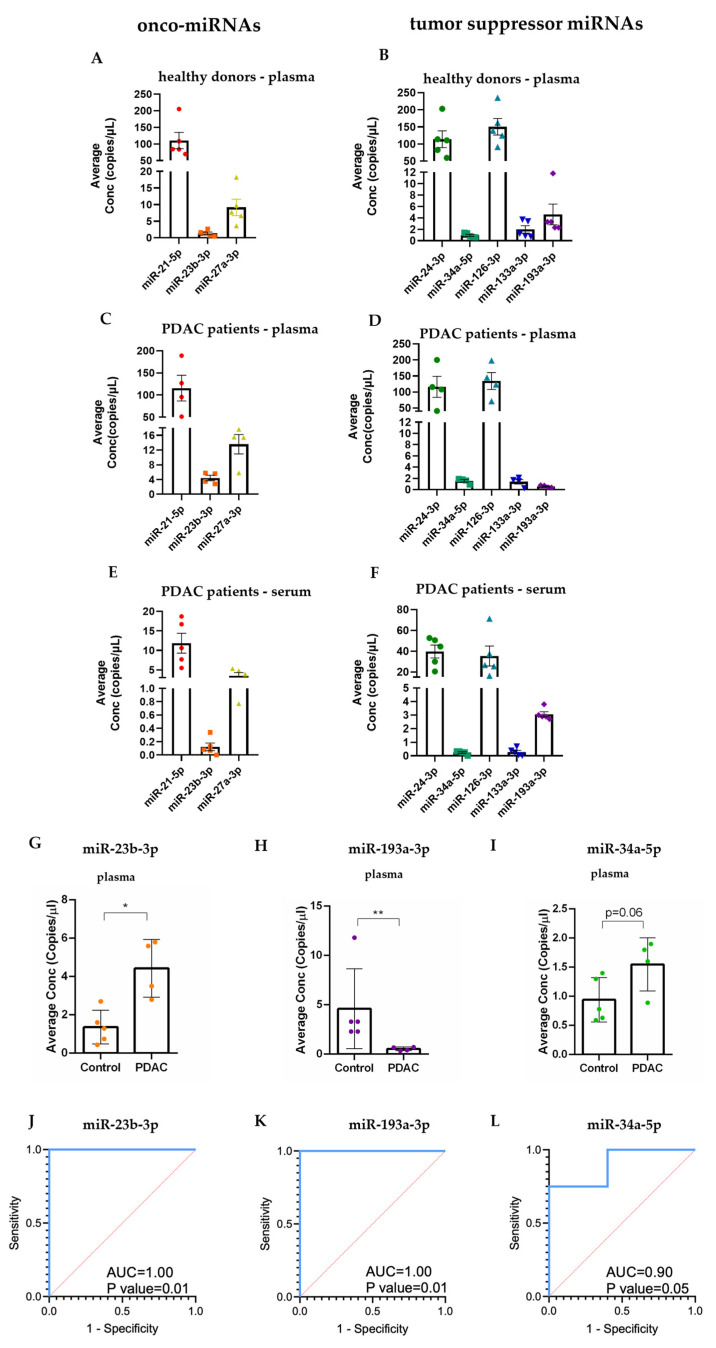
Quantification of circulating levels of selected miRNAs by ddPCR. (**A**,**B**) Levels of circulating onco-miRNAs and tumor suppressor miRNAs in plasma of healthy donors used as the controls (CTR04-08), (**C**,**D**) plasma of PDAC patients (EXOPANC03-06), (**E**,**F**) serum of PDAC patients (EXOPANC07-11). (**G**) Focus on the circulating levels of miR-23b-3p, (**H**) miR-193a-3p, (**I**) miR-34a-5p in plasma of healthy donors and PDAC patients. Histograms are the means of absolute quantification (concentration expressed as copies/µL), bars are SEM. * *p* < 0.05, ** *p* < 0.01; (**J**–**L**) ROC curves of circulating plasma miRNAs detected and quantified by ddPCR in EXOPANC patients (n = 4) and controls (n = 5). Blue ROC curves indicate significant AUCs: (**J**) miR-23b-3p (1.00), (**K**) miR-193a-3p (1.00), and (**L**) miR-34a-5p (0.90, with 95% confidence interval 0.77–1.00). Sensitivity and 1-Specificity are represented for all of the obtained copies/uL values. The red dotted line represents an AUC threshold of 0.50. AUC: area under the curve, ROC: Receiver operating characteristic.

With the aim of identifying miRNAs as potential molecular biomarkers with translational relevance, we quantified the levels of circulating miRNAs known to have oncogenic or tumor suppressing functions in PDAC and/or in other malignancies [21,41,42,43]. In particular, we performed the absolute quantification of the circulating onco-miRNAs (miR-21-5p, miR-23b-3p, miR-27a-3p) and tumor suppressor miRNAs (miR-24-3p, miR-34a-5p, miR-126-3p, miR-133a-3p and miR-193a-3p) using ddPCR. We analyzed a small cohort of samples to acquire informative, albeit preliminary, evidence for the selection of miRNAs for future prospective studies in a larger cohort. Thus, the levels of circulating miRNAs were measured in the plasma of 5 healthy donors and 4 PDAC patients and in the serum of 5 additional PDAC patients. Some general observations were made: (i) the selected miRNAs were detectable in the circulation of both patients and controls at varying levels; (ii) miR-21-5p, miR-24-3p and miR-126-3p were the most abundant in plasma of healthy controls (Figure 4A,B), PDAC patients (Figure 4C,D) and in serum of PDAC patients (Figure 4E,F); (iii) the miRNAs displayed lower levels in serum compared to plasma, suggesting plasma to be a more suitable material for circulating miRNA-based biomarker analysis; (iv) the trend of circulating levels of the miRNAs was superimposable in the plasma of healthy donors (Figure 4A,B) and in the serum of PDAC patients (Figure 4E,F). Focusing on the circulating plasma levels, miR-23b-3p showed higher average levels in plasma of PDAC patients compared to the healthy controls (Figure 4G,I). We take into consideration another circulating miRNA, miR-34a-5p, which showed an increase in EXOPANC patients with a less significative impact compared to the miR-23b-3p. On the contrary, miR-193a-3p exhibited lower average levels in PDAC patients compared to healthy donors (Figure 4H). 

Next, ROC analysis was performed to define the biomarker sensitivity and specificity and the same miRNAs were identified as the best performing biomarkers (Figure 4J–L and Figure S3A,B). These results may suggest that circulating miR-23b-3p, miR-193a-3p, and miR-34-5p should be considered in a future prospective study to investigate their plasmatic levels.

#### Pancreatic Cancer Cell Lines Exhibit Intracellular High Level of miR-23b-3p and Low Levels of miR-193a-3p

To further clarify the significance of the trend level variations of plasma circulating miR-23b-3p and miR-193a-3p in healthy individuals and PDAC patients, we extended the expression level analysis to a panel of human normal and pancreatic cancer cell lines, as well as their cognate sEVs that were already tested for the first subset of miRNA using RT-qPCR (Figure 2A–D). The expression levels of miR-23b-3p in normal and pancreatic cancer cell lines supported its implication as a tumor-promoting ncRNA as its levels were upregulated in three out of four cell lines with only one exception (MIA PaCa-2) (Figure S3C) and vesicle-associated miR-23b-3p showed the same trend. On the contrary, miR-193a-3p expression levels were downregulated in three out of four cell lines (not in PANC-1), supporting a tumor suppressor behavior (Figure S3D) while the levels of miR-193a-3p in sEVs secreted by the cell lines included in this study exhibit a general trend of variability (Figure S3E,F).

### 2.5. Different Peripheral Blood-Derived Circulating and Vesicle-Associated miRNAs Were Selected as Candidate Biomarkers for PDAC Patients

**Figure 5:**
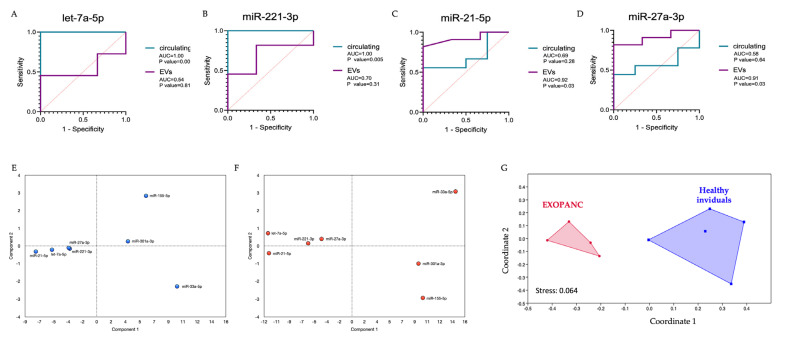
ROC curves with respective AUCs and *p* values of miRNAs (**A**) let-7a-5p, (**B**) miR-21-5p, (**C**) miR-221-3p, (**D**) miR-27a-3p quantified by RT-qPCR. In violet are the ROC curves of sEV-associated miRNAs detected in EXOPANC patients (n = 11) and controls (n = 3). In green are the ROC curves of circulating miRNAs extracted in EXOPANC patients (n = 9) and controls (n = 4). Sensitivity and 1-Specificity are represented for all of the obtained deltaCt values. The red line represents an AUC threshold of 0.50. (**E**,**F**) PCA analysis on circulating miRNAs in (**E**) healthy controls (blue dots) and (**F**) EXOPANC patients (red dots). (**G**) Non-metric multidimensional scaling (nm-MDS) analysis of miR-23b-3p, miR-193a-3p, and miR-34a-5p circulating miRNAs. On the left, the cluster is composed by pancreatic cancer patients (pink), and on the right healthy individuals (blue).

After selecting a few potential molecular biomarker candidates with translational relevance that may be considered in a future prospective study, we use the ROC curves to compare the results obtained from circulating and vesicle-related miRNAs. For the first subset of miRNAs measured with RT-qPCR, the sensitivity and specificity of the circulating candidates were different from the vesicle-associated selected miRNAs. Indeed, selected circulating let-7a-5p and miR-221-3p did not show significant results when analyzed in the vesicular fraction (Figure 5A,B). Similarly, miRNAs selected in the vesicular fraction, such as sEV-miR-21-5p and miR-27a-3p, did not show any significative variations in the circulating component (Figure 5C,D). Then, with the aim to understand whether the subset of miRNAs could have a role as biomarkers, a multiparametric analysis was performed. In particular, regarding the RT-qPCR data, the mean of deltaCt values of each sample obtained from PDAC patients and healthy individuals normalized with the reference miRNA were compared using a principal component analysis (PCA). Results from circulating miRNAs (Figure 5E,F) display two ordination plots, and it is possible to note that the two miRNAs, let-7a-5p and miR-221-3p, were selected for their different levels between pancreatic cancer patients and healthy individuals in fold-change analysis (Figure 3C), and cluster closely with each other and with two other miRNAs, miR-21-5p and miR-27a-3p. These miRNAs showed a ROC curve significant value in the vesicular fraction (Figure 5C,D), and both in the PCA representing the control samples (Figure 5E) and in the PCA representing the pancreatic cancer patients (Figure 5F). Therefore, a Manova test was conducted using (i) the data values of all the miRNAs analyzed in the subset; (ii) the data of the clustered miRNAs let-7a-5p, miR-221-3p, miR-21-5p, and miR-27a-3p and (iii) the two circulating miRNAs let-7a-5p and miR-221-3p (Table 2). All three sets of samples showed a significative *p*-value, indicating that they are efficient in differentiating pancreatic cancer patients from healthy individuals. Similarly, the three sets of data were utilized to perform linear discrimination analysis (LDA). The results of this analysis revealed that the samples correctly classified in the LDA model generated with the expression data of all miRNAs were 92.31% (Table 2). This percentage increased to 100% when the 4 miRNAs let-7a-5p, miR-221-3p, miR-21-5p, and miR-27a-3p or the couple of miRNAs let-7a-5p and miR-221-3p were considered (Table 2), indicating that these two groups of miRNAs may constitute a strong predictive model for differentiating pancreatic cancer patients from healthy individuals. PCA was also repeated for sEVs-associated miRNAs (Figure S4A,B). However, in this case, the two miRNAs that had shown significance in previous ROC analyses appeared distant from each other and from other miRNAs and the same tests (Manova and LDA tests) showed that, when analyzing the entire dataset of miRNAs, the *p*-value from the Manova test was not significant (Table 2) and the percentage of correct predictions was 71.43% (Table 2). Only when considering the two vesicular miRNAs, miR-155-5p and miR-27a-3p, the Manova test showed a significant *p*-value (<0.05) and the percentage of correct predictions according to the LDA test was 78.57% (Table 2).

Regarding the second subset of circulating miRNAs obtained with ddPCR, a non-metric multidimensional scaling (nm-MDS) analysis was performed on circ-miR-23b-3p, circ-miR-34a-5p, and circ-miR-193a-3p derived from plasma (Figure 5G). These three miRNAs were selected from ROC curves analysis for their significant diagnostic value. The proposed approach on the data of the 3 miRNAs constructs two different and distinct clusters of data, the cluster in pink includes data from patients (left), while the cluster in blue includes data from healthy individuals (right) (Figure 5G). Therefore, considering these 3 miRNAs, patients and the controls form two distinct clusters, making possible the distinction between the two groups. These results demonstrate that taking into consideration a combination of the selected circulating and vesicle-associated miRNAs could improve the relevance of miRNAs as molecular biomarkers.

## 3. Discussion

PDAC is a deadly malignancy worldwide. Patients usually do not exhibit specific symptoms in early cancer phases, making the diagnostic rate at localized stage very low [2,3]. Serum markers that are widely used in other kinds of neoplasia, such as CA19-9 or CEA, lack sensitivity and specificity in PDAC since their levels are often normal during the early stages of the disease or are falsely high in individuals with other extra-pancreatic malignancies and benign hepatopancreaticobiliary conditions [44]. Hence, the identification of early, non-invasive diagnostic biomarkers is crucial for the diagnosis, prognosis, and choice of treatments. In this regard, the extracellular release and stability in the already described deregulation of miRNAs in PDAC cells led to focus in the research to understand their potential as circulating diagnostic and prognostic tools [22,23].

Currently, gemcitabine is one of the most used PDAC chemotherapeutic drugs, but its efficiency is limited due to the development of resistance. In this preliminary study, with the aim of identifying miRNAs as potential molecular biomarkers with translational relevance, we have first selected a set of miRNAs suggested to be possibly involved in gemcitabine chemoresistance and we measured their cellular expression and their levels in sEVs from a panel of PDAC cell lines and from serum/plasma of a small case series of PDAC patients compared to respective non-tumor controls. Our results show a dysregulation of the levels of a subset of miRNAs taken into consideration. In particular, low levels of miR-155-5p in both cells and sEVs of PDAC cell lines compared to non-tumor cells were described. Firstly, these results indicate that miR-155 levels in sEVs may reflect cellular miR-155 expression. This concordance was also reported between the levels of miR-155 in PDAC tumor tissue and the corresponding EVs [45]. Therefore, these data suggest that vesicle-associated miR-155 levels may represent a promising clinical tool. Furthermore, miR-155 is considered an oncogenic miRNA in PDAC, involved in tumor progression and resistant to gemcitabine [21,46]. Indeed, it was reported that the gemcitabine exposure of PDAC cells increases miR-155 levels with two different effects: induction of exosome secretion to deliver the miR-155 into other PDAC cells and chemoresistance [45]. Here, we described low levels of sEVs-associated miR-155-5p in pancreatic cancer patients’ samples not treated with gemcitabine compared to the controls, which indicates that this miRNA is downregulated and less secreted. This dysregulation could indicate lower gemcitabine chemoresistance as increased miR-155 levels were reported after gemcitabine exposure [45]. Interestingly, we observed unchanged intracellular miR-155 levels and a decrease in the same vesicle-associated miRNA in gemcitabine-resistant YAPC cells after treatment. These data confirm the expected behavior of chemoresistant cells and add novel information about the amount of this miRNA in sEVs from chemoresistant cells. Indeed, YAPC, derived from ascitic fluid and representing aggressive and advanced stage cancer cells, are surprisingly characterized by reduced levels of vesicular miR-155. As reported in the literature, malignant ascites comprise not only tumor cells, but also many other non-tumor cell types and acellular components, which produce a unique microenvironment that can modify the neoplastic properties of tumor cells [47]. Thus, we can speculate that YAPC cells, as part of the pancreatic tumor microenvironment, secrete fewer EVs compared to pancreatic mesenchymal cells. Further studies are needed to understand the role of EV secretion from ascitic cell lines. Moreover, this aspect should be further investigated in a larger cohort of patients and, in particular, before and after gemcitabine treatment, trying to establish if, in patients with low levels of vesicle-associated miRNA at diagnosis, target therapy to maintain or reduce miR-155 low levels could be useful to contrast and reduce resistance to treatment. 

In pancreatic cancer cells, vesicle-associated miR-27a was reported to promote angiogenesis and induce proliferation and invasion [48]. Low levels of cellular miR-27a-3p were detected in pancreatic cancer cells, while the level of miR-27a-3p was slightly increased in the plasma of PDAC patients compared to healthy donors (Figure 4A,C) (13.6 ± 2.6 vs. 9.2 ± 2.5 copies/µL). Interestingly, the level of miR-27a-3p in sEVs of PDAC patients was also reported to be higher compared to healthy subjects. Therefore, miR-27a-3p showed the same trend as both the circulating plasma miRNA and plasma vesicle-associated miRNA. This may indicate a prominent role for this miRNA outside the cells making it a potential non-invasive diagnostic biomarker. 

Furthermore, the selected second set of miRNAs included miRNAs with an oncogenic role, or generally upregulated in human tumors, and miRNAs with a tumor suppressor function, or generally downmodulated in human malignancies. For instance, the onco-miR-21-5p is known to be upregulated in PDAC and to promote cell proliferation, cell cycle, tumor growth in vivo, and the inhibition of apoptosis in vitro [49]. The miR-23b-3p directly targets the oncosuppressor PTEN (Phosphatase and TENsin homolog deleted on chromosome 10), its overexpression promotes the tumor growth and liver metastasis of pancreatic cancer xenografts developed in mouse models [50]. Furthermore, the onco-miR-27a-3p is upregulated in pancreatic cancer tissue compared to adjacent non-tumor tissues and acts as an oncogene by regulating colony formation, cell proliferation, and migration [51]. The tumor suppressor miR-24-3p and miR-193a-3p are down-modulated in PDAC, modulating the aggressive properties of PDAC in vitro and in vivo [49]. Also, miR-34a-5p is down-modulated in pancreatic cancer patients and low miR-34a-5p expression level was reported to correlate with worse prognosis compared to patients with high expressions of the same miRNA in terms of overall survival [34]. The oncosuppressor miR-126-3p has been used to generate a signature of 7 miRNAs down-modulated in pancreatic cancer able to predict the prognosis identified by bioinformatic tools [52]. Finally, miR-133a-3p is generally down-regulated in several malignancies including gastric cancer, bladder cancer, oral squamous cell carcinoma, and non-small cell lung cancer [7,53,54,55].

The detection of miR-23b-3p and miR-193a-3p in plasma/serum, and generally in human body fluids, has been very poorly reported. The levels of miR-23b-3p are high in the sera samples and high levels of miR-23b-3p were also detected in the sEV from the sera of pancreatic cancer patients [50,56]. It was also reported that miR-23b-3p was significantly upregulated in the saliva of pancreatic cancer patients compared to controls [57]. Also, miR-193a-3p expression levels were significantly lower in PDAC tissues compared to non-cancerous tissues [58]. However, to the best of our knowledge, no data have been reported on miR-193a-3p in the circulation, body fluids, or sEVs. In this report, considering circulating plasma levels, miR-23b-3p showed higher levels in PDAC patients compared to the healthy controls, while miR-193a-3p showed lower average levels. However, the determination of miR-23b-3p and miR-193a-3p levels in sEVs from the conditioned media of pancreatic cancer cells and from the plasma/serum of PDAC patients did not show significant differences. This may suggest that in a future larger study, the determination of circulating plasma levels of miR-23b-3p and miR-193a-3p will be more promising to verify their role as potential translational molecular biomarkers that can differentiate PDAC patients from healthy controls. 

In conclusion, despite the small cohort of analyzed patients, our results highlighted a potential clinical significance of vesicle-associated miR-155-5p and miR-27a-3p, circulating let-7a-5p, miR-221-3p, miR-23b-3p, and miR-193a-3p, allowing for the identification of a signature to distinguish pancreatic cancer patients from healthy controls. Moreover, this dysregulation suggests that these miRNAs should be considered in a future prospective study in a larger cohort of PDAC cases and healthy controls to explore their diagnostic value as molecular biomarkers.

## 4. Materials and Methods

### 4.1. Case Series and Samples

Blood samples were collected using commercial collection tubes from pancreatic cancer patients and healthy donors. A total of 11 patients were enrolled between 2022 and 2023 within the frame of the EXOPanc (role of exosomes in pancreatic cancer progression) study, approved by the Area Vasta Emilia Centro Ethical Committee. All cases were diagnosed at the S. Orsola-Malpighi Hospital, Bologna, Italy, the mean (±standard deviation) age of patients was 69.1 ± 10 years (range from 59 to 85 years). All patients were diagnosed with poorly differentiated pancreatic adenocarcinoma G3 (histologic grading system) [59,60]. Four patients were diagnosed with stage II/III (EXOPANC03, 06, 10, 11), while the majority of cases presented stage IV pancreatic cancer and distant metastasis to other areas of the body, such as the liver. The detailed characteristics of patients’ cohort are summarized in Table 1. An alpha-numeric code (from EXOPANC01 to EXOPANC11 for PDAC patients and from CTR01 to CTR08 for healthy donors) was assigned to maintain anonymity. The characteristics of healthy donors are listed in Table S3. Plasma or serum were collected, together with the informed consent obtained in compliance with the Helsinki Declaration. Internal review board protocols were followed for the collection of samples. Serum was collected for patients EXOPANC01, 02, 07-11, and healthy donors CTR01-03: after collection, blood samples were maintained for 30 min at room temperature to form a clot, then centrifugated at 1100× *g* for 15 min at 4 °C. The upper clear fraction (serum) was collected in 0.5-mL aliquots and immediately stored at −80 °C. For patients EXOPANC03-06 and healthy donors CTR04-08, plasma separation was obtained from blood collected in EDTA, samples were centrifugated at 3000× *g* for 10 min at 4 °C. The upper fraction (plasma) was collected in 0.5-mL aliquots and stored at −80 °C. The use of plasma or serum from the different individuals was due to the availability of the sample. 

### 4.2. Isolation of miRNA from Small EVs in Serum/Plasma Samples

Total RNA including mRNA, miRNA, and other noncoding RNAs, was isolated from the sEVs, starting from 200 μL of serum/plasma. Samples were diluted with 800 μL of phosphate-buffered saline (PBS) to reduce fluid viscosity and centrifuged at 2000× *g* at 4 °C for 30 min. Pellets consisting of cell debris were discarded while supernatants were collected and centrifuged at 12,000× *g* at 4 °C for 45 min to remove apoptotic bodies, mitochondrial fragments, cell debris, and large vesicles (mean size > 200 nm). Serum/plasma were then filtered to exclude particles larger than 0.22 μm using syringe filters in order to select for small EVs. The samples were then processed using the exoRNeasy Midi Kit (Qiagen, Cat#: 1116083, Hilden, Germany), following the manufacturer’s instructions. Briefly, samples were added onto an exoEasy spin column to isolate sEVs. QIAzol Reagent (Qiagen, Hilden, Germany) was used for the lysis and elution of sEVs. Chloroform was added to the QIAzol eluate and up to 400 μL of the aqueous phase was recovered; ethanol was added and the solution was transferred to the RNeasy MinElute Spin Columns. Total RNA, included miRNA, was collected using RNAse-free water, producing elutes of approximately 12 μL. An RNA synthetic spike-in mix, including UniSp2, UniSp4, and UniSp5 (Qiagen, Hilden, Germany), was added to each sample before isolation as the control to detect differences in extraction efficiency between samples.

### 4.3. Cell Lines

A set of 4 different human PDAC cell lines were used. MIA PaCa-2 (Cellosaurus Research Resource Identifiers RRID:CVCL_0428 [61]) and PANC-1 (RRID:CVCL_0480 [61]) cells were grown in DMEM (Euroclone) while YAPC (RRID:CVCL_1794 [61]) and BxPC-3 (RRID:CVCL_0186 [61]) cells were maintained in RPMI-1640 medium (Euroclone). Media were supplemented with 10% FBS, 2 mM glutamine, 100 U/mL penicillin, and 10 mg/mL streptomycin (Euroclone). The immortalized epithelial cell line derived from normal human pancreatic duct epithelial cells H6c7 (RRID:CVCL_0P38 [61]) was cultured in Keratinocyte SFM, + 2.5 µg EGF human recombinant + 25 mg bovine pituitary extract (Gibco Cat#: 10724-011 and 37000-015) supplemented with 1× Antibiotic-Antimycotic (Gibco Cat#: 15240-062). All the cell lines were grown in a 5% CO_2_ incubator at 37 °C. MIA PaCa-2 and PANC-1 were kindly provided by Professor Miriam Martini (University of Turin, Turin, Italy) [62], while YAPC, BxPC-3, and H6c7 were a gift from Doctor Loredana Moro (New York University, New York, NY, USA and National Research Council Bari, Bari, Italy) [63]. 

#### 4.3.1. Cell Treatment and Viability Measurement in Presence of Gemcitabine

Cell viability for YAPC, BxPC-3, MIA PaCa-2 and PANC-1 cell lines was measured by the MTT (3-(4,5-dimethylthiazol-2-yl)-2,5-diphenol tetrazolium bromide) assay. Conversion of MTT by mitochondrial succinate dehydrogenase was used as an indicator for cell density determination as already described [64,65]. Cells were seeded in 96-well plates (Corning) (5 × 10^3^ cells/well) in a complete medium; after 24 h cells were washed twice in PBS and incubated in different media. To evaluate cytotoxicity, different concentrations of gemcitabine (Cayman Chemical) were used (25 nM, 75 nM, 250 nM, 750 nM, 1 µM). Plates reading was performed on a Multilabel Plate Reader (Victor X5, PerkinElmer, Waltham, MA, USA) at 570 nm. The half-maximal effective concentration (EC50) value is used as a measure of the drug potency. EC50 was calculated after 72 h as the concentration that results in a 50% decrease in the number of cells compared to that of the untreated cells using the GraphPad Prism software (Version 6.0, GraphPad, San Diego, CA, USA). Regarding gemcitabine treatment, cells were seeded in complete medium. After 24 h, cells were washed 3 times with PBS to completely remove FBS and successively were incubated with medium containing 10% exosome-free FBS, 2 mM glutamine, 100 U/mL penicillin, and 10 mg/mL streptomycin and 20 nM of gemcitabine or without gemcitabine for 72 h. The EC50 value of the most sensitive cell line, MIA PaCa-2 cells, was used for the treatment of all PDAC cell lines. Intracellular and vesicle-associated miRNA were isolated.

#### 4.3.2. Isolation of miRNAs from Small EVs in Cultured Cells

Cells presenting a confluence around 70%, were washed 3 times with PBS to completely remove FBS and were successively incubated with medium containing 10% exosome-free FBS, 2 mM glutamine, 100 U/mL penicillin, and 10 mg/mL streptomycin. After 48 h, the conditioned medium was taken and centrifuged two times at 300× *g* for 10 min at 4 °C to pellet cells. The supernatant was collected and centrifuged at 16,500× *g* for 20 m in at 4 °C to pellet apoptotic bodies and cell debris. The supernatant was then filtered through a 0.22 μm syringe filter and total miRNA extraction was performed using an exoRNeasy Midi Kit (Qiagen, Cat#: 1116083, Hilden, Germany) for the isolation of total RNA from exosomes and other EVs, using the protocol provided by Qiagen, briefly reported in Section 4.2.

#### 4.3.3. Purification of miRNA from Cells

In parallel, for each cell line, 2 × 10^6^ cells were collected and miRNA extraction was performed using miRNeasy Mini Kit (Qiagen Cat#: 217004, Hilden, Germany) following manufacturer’s instructions. Briefly, the cell pellet was lysed using QIAzol Lysis Reagent and homogenized. Chloroform was added and less than 350 μL of the aqueous phase was used with the RNeasy Mini spin column. Total miRNA was eluted in 30 μL of RNase-free water. RNA quantity was assessed using Nanodrop (Thermo Fisher Scientific, Inc., Waltham, MA, USA).

### 4.4. Prediction of miRNA Targeting ABCB1 Amplicon

The prediction of miRNAs, which potentially target *ABCB1* amplicon [13], was performed using MIENTURNET (MicroRNA ENrichment TURned NETwork) [36] and miRTargetLink 2.0 [40].

The MIENTURNET http://userver.bio.uniroma1.it/apps/mienturnet/ (accessed on 14 March 2024) analysis parameters were used as follows: MicroRNA/Genes Network, miRTarBase (an experimentally validated miRNA-target interaction database); default parameters, i.e., threshold for the minimum number of miRNA-target interactions: 2; threshold for the adjusted *p*-value (FDR): 1. Filter by Evidence categories: Strong and Weak [36]. miRTargetLink 2.0 https://ccb-compute.cs.uni-saarland.de/mirtargetlink2/ (accessed on 14 March 2024) analysis parameters were used as follows: (Edit network) Unidirectional search; Interaction landscape for a single gene symbol; miRNA targets: strong validated and weak validated; Min shared targets: 1 [40] (Table S2). Furthermore, an analysis of the current available published literature was performed [25] to obtain the selected set of miRNA including hsa-let-7a-5p, hsa-miR-301a-3p, and hsa-miR-27a-3p.

### 4.5. Expression of Predicted miRNAs with RT-qPCR

The miRNAs levels were measured using miRNA assays (miRCURY miRNA Assay, Qiagen, Hilden, Germany; hsa-let-7a-5p Cat#: YP00205727, hsa-miR-191-5p Cat#: YP00204306, hsa-miR-103a-3p Cat#: YP00204063, hsa-miR-16-5p Cat#: YP00205702, hsa-miR-1-3p Cat#: YP00204344, hsa-miR-21-5p Cat#: YP00204230, hsa-miR-33a-5 Cat#: YP00205690, hsa-miR-129-5p Cat#: YP00204534, hsa-miR-155-5p Cat#: YP02119311, hsa-miR-221-3p Cat#: YP00204532, hsa-miR-301a-3p Cat#: YP00205601, hsa-miR-27a-3p Cat#: YP00206038, U6 snRNA Cat#: YP02119464, UniSp6 Cat#: YP00203954, cel-miR-39-3p Cat#: YP00203952, UniSp2 Cat#: YP00203950, UniSp4 Cat#: YP00203953, UniSp5 Cat#: YP00203955). The levels of miRNAs were analyzed from cell and sEVs in cultured cells and in plasma/serum (vesicle-associated and circulating miRNAs) of patients diagnosed with PDAC and from healthy individuals using RT-qPCR. The miRCURY LNA™ SYBR^®^ Green PCR Kit (Qiagen, Cat#: 339347, Hilden, Germany) was used for the miRNA relative quantification analysis. An RNA synthetic spike-in mix including UniSp6 and cel-miR-39-3p (cDNA synthesis control) were used for monitoring successful reverse transcription. Each spike-in was added to every reverse transcription reaction to determine the effectiveness of the process. For miRNA extracted from cells, 10 ng of total RNA were reverse transcribed to cDNA using the miRCURY LNA RT Kit (Qiagen, Cat#: 339340, Hilden, Germany) and diluted (60×). For the total RNA extracted from serum/plasma and from the EVs, following the manufacturer’s protocol, the amount of RNA equivalent to that isolated from 16ul sample was used for the RT reaction with miRCURY LNA RT Kit (Qiagen, Cat#: 339340, Hilden, Germany) and diluted (30×). Then, cDNAs were mixed with miRCURY LNA SYBR Green PCR Kits (Qiagen, Cat#: 339345, Hilden, Germany) and the qPCR reaction was performed using the Bio-Rad CFX96 Real Time PCR Machine (Bio-Rad, Hercules, CA, USA).

### 4.6. Isolation of Circulating RNA from Plasma and Serum and ddPCR Analysis

#### 4.6.1. RNA Isolation from Plasma and Serum and Reverse Transcription (RT)

Total RNA was isolated from 200 µL of plasma or serum using miRNeasy Mini Kit (Qiagen), according to the manufacturer’s instructions as previously reported [66]. For miRNAs analysis, cDNA was synthesized from 2.5 µL of RNA from plasma/serum, in a 7.5 µL reaction volume, using the TaqMan microRNA Reverse Transcription Kit components (Thermo Fisher Scientific) and the stem-loop primer for miR-21-5p (Thermo Fisher Scientific; Assay ID 000397), miR-23b-3p (Thermo Fisher Scientific; Assay ID 000400), miR-24-3p (Thermo Fisher Scientific; Assay ID 000402), miR-27a-3p (Thermo Fisher Scientific; Assay ID 000408), miR-34a-3p (Thermo Fisher Scientific; Assay ID 002316), miR-126-3p (Thermo Fisher Scientific; Assay ID 002228), miR-133a-3p (Thermo Fisher Scientific; Assay ID 002246), and miR-193a-3p (Thermo Fisher Scientific; Assay ID 002250) [41]. The RT reaction took place initially at 16 °C for 30 min, then at 42 °C for an additional 30 min, and was concluded with inactivation at 85 °C for 5 min using a T100 Thermal Cycler (Bio-Rad Laboratories).

#### 4.6.2. Droplet Digital PCR Workflow

QX200 Droplet Digital PCR System (Bio-Rad Laboratories) was employed to carry out this experiment and the synthesized cDNA was used as a template for the ddPCR experiments. ddPCR was performed according to the ddPCR Supermix for Probes (Bio-Rad Laboratories) protocol, as previously described [67]. Briefly, 1.33 µL of the cDNA obtained using TaqMan microRNA Reverse Transcription Kit, were prepared for amplification in a 20 µL reaction volume containing 2× ddPCR Supermix for Probes (Bio-Rad Laboratories), 20× TaqMan assay (Thermo Fisher Scientific) specific for miR-21-5p, miR-23b-3p, miR-24-3p, miR-27a-3p, miR-34a-3p, miR-126-3p, miR-133a-3p, miR-193a-3p, and water. Each ddPCR assay mixture (20 µL) was loaded into a disposable droplet generator cartridge (Bio-Rad). Next, the wells specifically designated for oil were filled with 70 µL of droplet generation oil for probes (Bio-Rad). The cartridge was then placed inside the QX200 droplet generator (Bio-Rad). When droplet generation was completed, the droplets were transferred to a 96-well PCR plate using a multichannel pipette. The plate was heat-sealed with foil and placed in a T100 Thermal Cycler (Bio-Rad Laboratories). A negative control (NC) and a positive control (PC) were also used. Concentration data for miR-21-5p, miR-23b-3p, miR-24-3p, miR-27a-3p, miR-34a-3p, miR-126-3p, miR-133a-3p, and miR-193a-3p levels were obtained using QuantaSoft Software (Bio-Rad Laboratories) and was expressed as copies/µL.

### 4.7. Data Analysis and Statistics

For the statistical evaluation of the RT-qPCR analysis, data were controlled for normal distribution using the Shapiro–Wilk test. Qiagen GeneGlobe Data Analysis Center https://geneglobe.qiagen.com/us/analyze (accessed on 10 April 2024) and in particular, the miRCURY LNA miRNA PCR Assays tool was used to detect dysregulated miRNAs. The levels of miRNAs were determined according to the Ct (Cycle threshold) values. The assay cut-off was 40 cycles. At the end of the RT-qPCR reaction, raw Ct values and melting curves were exported for the analyses. Raw Ct values were normalized by averaging of internal controls. A small panel of 4 well-known references (U6 snRNA, miR-191-5p and miR-103a-3p, miR-16a-5p) were initially selected for the data normalization. The use of Normfinder software and geNORM algorithm [68,69] to calculate the stability value and to select the most suitable normalizer, identified miR-16-5p as the most stable endogenous reference gene for both intracellular and sEVs dataset in cell lines and patients and miR-103a-3p in the circulating miRNAs dataset. The deltaCt values of samples were calculated using the respective reference miRNAs. The relative expression of the examined miRNAs between the two groups was determined by the ΔΔCT method, taking into account the mean values of all ΔCT within a group. 

Two-fold-changes in miRNA rates (2 ≤ FoldChange ≤ 0.5) between the two groups were defined as statistically relevant. Statistical relevance of the miRNA levels between the two groups was analyzed by two-tailed unpaired t-test. The Mann–Whitney test was used for comparing datasets with unequal variance. ROC curves and respective statistical analyses were performed using GraphPad Prism 8.4.3. A *p*-value ≤ 0.05 was considered to indicate a statistically significant result. GraphPad Prism software (Version 6.0) was used to generate the graphs of deregulated miRNAs. Different comparisons of differential expression were performed: cancer cell lines (BxPC-3, YAPC, MIA PaCa-2, PANC-1) vs. non cancer cell lines (H6c7), gemcitabine treated cells vs. untreated cells, and PDAC patients vs. healthy controls. All experiments are represented as the mean ± SEM. 

The discriminant analysis (LDA) (Multivariate > Ordination > discriminant analysis), the ordinations plots (Principal Component Analysis (PCA) and the non-metric multidimensional scaling (NM-MDS)) (Multivariate > Ordination > Principla components; Multivariate > Ordination > Non-Metric MDS), the MANOVA tests (Multivariate > tests > MANOVA) were carried out with PAST v.4 [70].

## Figures and Tables

**Table 1:** Clinical characteristics of patients with pancreatic cancer.

**Characteristics**	
Gender (F/M)	3/8
Age, mean years (range)	69.1 (59–85)
Size of lesion, median mm (range)	39.6 (15–59)
Tumor stage, n (%)	
II	1 (9.1%)
III	3 (27.3%)
IV	7 (63.6%)
Lesion location, n (%)	
Pancreatic head	7 (63.6%)
Pancreatic body	3 (27.3%)
Pancreatic tail	1 (9.1%)
Distant metastases, n (%)	
M0	4 (36.4%)
M1	7 (63.6%)

Abbreviations: F/M, Female/Male; M, distant metastases.

**Table 2:** Significative differences between PDAC and the control group according to the Manova test and percentage of data that the LDA model correctly classifies.

	Circulating		sEV	
	Manova *p*-Value ^1^	LDA (% Correctly Classified) ^2^	Manova *p*-Value ^1^	LDA (% Correctly Classified) ^2^
All miRNAs	0.0117 *	92.31%	0.3690	71.43%
let-7a-5p, miR-21-5p, miR-221-3p, miR-27a-3p	0.0006 *	100%	0.2093	64.29%
miR-27a-3p, miR-155-5p	0.8959	30.77%	0.0210*	78.57%
let-7a-5p, miR-221-3p	0.00002	100%	0,7119	57.14%

^1^ = Bonferroni-corrected *p*-value; ^2^ = Jackknife Test. * *p*  <  0.05.

## Data Availability

Datasets are available on request from authors.

## Supplementary Materials

The following supporting information can be downloaded at: https://www.mdpi.com/article/10.3390/ncrna10030029/s1. Figure S1: Intracellular and vesicle-associated miRNA relative expression evaluated by RT-qPCR. (A) Intracellular expression levels and (B) levels of selected vesicle-associated miRNAs in each PDAC cell line compared to the control H6c7 cells. Grand mean is shown; (C) Viability of the PDAC cancer cell lines during 72 h treatment with increasing gemcitabine concentrations. Data are presented as mean  ±  SD normalized to T0 (n  =  3; * *p* < 0.05; ** *p* < 0.01, *** *p* < 0.001); (D–E) Intracellular expression of selected miRNAs in BxPC-3 and (E) in PANC-1 cells after 72 h treatment with 20 nM gemcitabine (GEM) compared to the untreated cells. (F-G) Levels of selected sEV-miRNAs in the medium of BxPC-3 and PANC-1 cells after 72 h treatment with 20 nM gemcitabine (GEM) compared to untreated cells. Data are mean  +  SEM (** *p*  <  0.01); Data are mean  +  SEM (* *p* < 0.05; ** *p* < 0.01; *** *p* < 0.001). Figure S2: (A) Levels of selected vesicle-associated miRNAs in each pancreatic cancer patient (EXOPANC01-11). Fold-change between each EXOPANC patient compared to controls was reported; (B) Levels of selected circulating miRNAs in each pancreatic cancer patient (EXOPANC03-11). Fold-change between each EXOPANC patient compared to the controls was reported; (C-D) ROC curves with non-significant AUC. (C) Respective AUCs and *p* values of sEV-associated miRNAs detected in EXOPANC patients and controls quantified by RT-qPCR; (D) ROC curves of circulating miRNAs quantified by RT-qPCR extracted in EXOPANC patients and controls. The red dotted line represents an AUC threshold of 0.50. Sensitivity and 1-Specificity are represented for all of the obtained deltaCt values. Figure S3: ROC curves with non-significant AUC. Respective AUCs and *p* values of miRNAs quantified by ddPCR. Sensitivity and 1-Specificity are represented for all the obtained copies/µL values. (A) ROC curves of circulating plasma miRNAs detected in EXOPANC patients and controls. (B) ROC curves of sEV-associated miRNAs extracted in EXOPANC patients and controls. The red dotted line represents an AUC threshold of 0.50. (C) Expression levels of miR-23b-3p and (D) miR-193a-3p in human normal and pancreatic cancer cell lines; (F) Levels of miR-23b-3p and (G) miR-193a-3p and in sEVs released in the media of pancreatic normal and cancer cells. Figure S4: PCA analysis on vesicle-associated miRNAs in (A) healthy controls (blue dots) and (B) EXOPANC patients (red dots). Table S1. Mienturnet Enrichment results as obtained from miRTarBase. Table S2. Relationships between miRNAs and target genes using the miRTargetLink 2.0 platform https://ccb-compute.cs.uni-saarland.de/mirtargetlink2/ (accessed on 14 March 2024). In orange the miRNAs identified using miRTargetLink 2.0. Table S3. Characteristics of healthy donors. Abbreviations: F/M, Female/Male.
