# Supplementary material for: Dysregulation of a Subset of Circulating and Vesicle-Associated miRNA in Pancreatic Cancer
Source: Noncoding RNA. 2024 May 1;10(3):29. doi: 10.3390/ncrna10030029 (PMC11130804; doi:10.3390/ncrna10030029)
Supplement: Supplementary file 1 [file ncrna-10-00029-s001.zip › ncrna-2903595-supplementary.pdf]

Article

Dysregulation of a Subset of Circulating and Vesicle-Associated miRNA in Pancreatic Cancer

Giulia Girolimetti [1], Iulia Andreea Pelisenco [2], Leonardo Henry Eusebi [3,4], Claudio Ricci [3,5], Beatrice Cavina [3,6], Ivana Kurelac [3,6], Tiziano Verri [1], Matteo Calcagnile [1], Pietro Alifano [7], Alessandro Salvi [2], Cecilia Bucci [7,*] and Flora Guerra [1]

1 Department of Biological and Environmental Sciences and Technologies (DiSTeBA), University of Salento, Via Provinciale Lecce-Monteroni 165, 73100 Lecce, Italy; giulia.girolimetti@unisalento.it (G.G.); tiziano.verri@unisalento.it (T.V.); matteo.calcagnile@unisalento.it (M.C.); flora.guerra@unisalento.it (F.G.)

2 Department of Molecular and Translational Medicine, University of Brescia, Viale Europa 11, 25123 Brescia, Italy; i.pelisenco@unibs.it (I.A.P.); alessandro.salvi@unibs.it (A.S.)

3 Department of Medical and Surgical Sciences (DIMEC), University of Bologna, Via Massarenti 9, 40138 Bologna, Italy; leonardo.eusebi@unibo.it (L.H.E.); claudio.ricci6@unibo.it (C.R.); beatrice.cavina2@unibo.it (B.C.); ivana.kurelac@unibo.it (I.K.)

4 Gastroenterology Unit, IRCCS Azienda Ospedaliero-Universitaria Di Bologna, Via Massarenti 9, 40138 Bologna, Italy;

5 Pancreatic Surgery Unit, IRCCS Azienda Ospedaliero-Universitaria Di Bologna, Via Massarenti 9, 40138 Bologna, Italy;

6 Centre for Applied Biomedical Research (CRBA), University of Bologna, Via Massarenti 9, 40138 Bologna, Italy

7 Department of Experimental Medicine (DiMeS), University of Salento, Via Provinciale Lecce-Monteroni 165, 73100 Lecce, Italy; pietro.alifano@unisalento.it (P.A.)

* Correspondence: cecilia.bucci@unisalento.it (C.B.); Tel.: +39-0832-298900

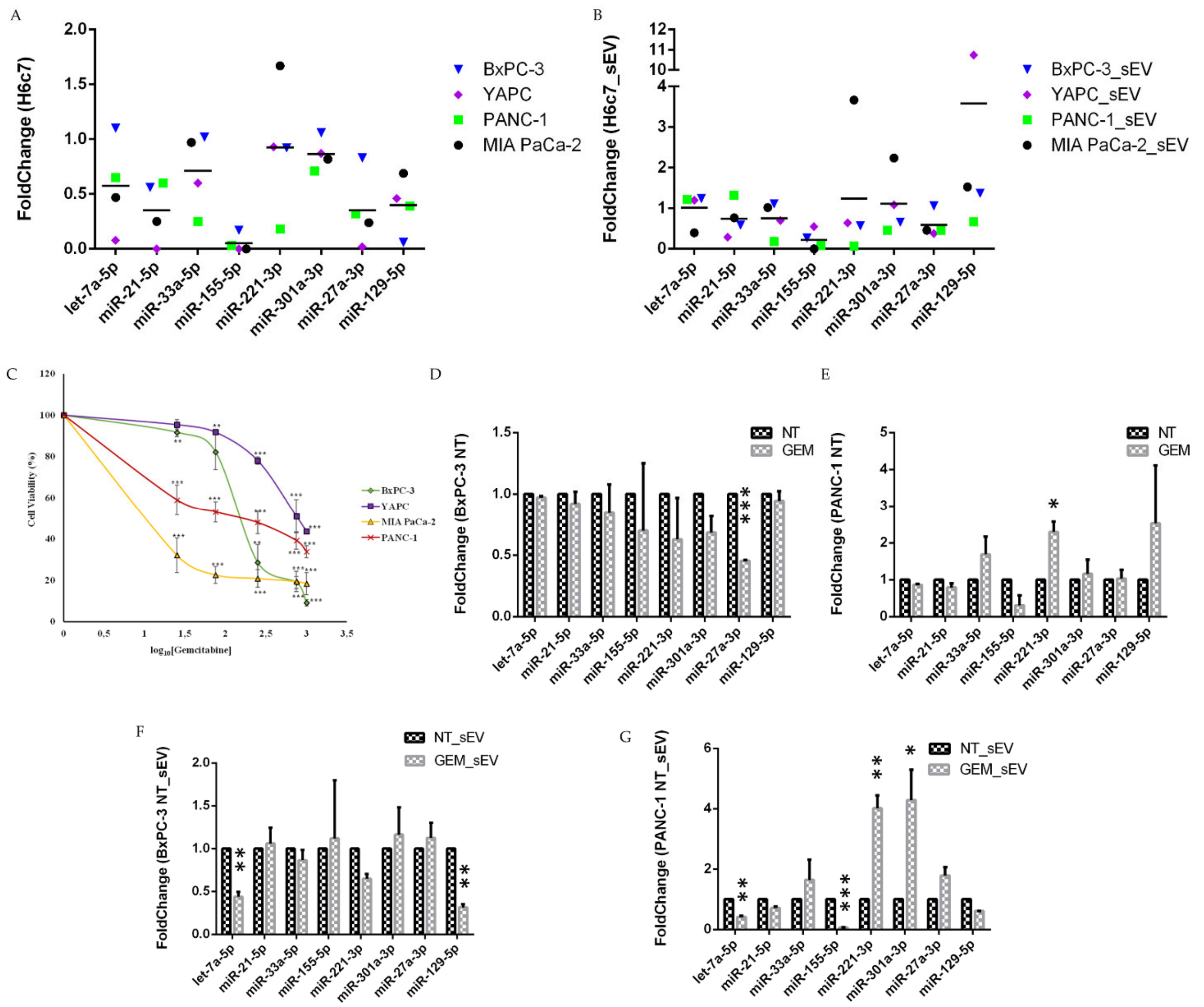

Figure S1. Intracellular and vesicle-associated miRNA relative expression evaluated by RT-qPCR. (A) Intracellular expression levels and (B) levels of selected vesicle-associated miRNAs in each PDAC cell line compared to the control H6c7 cells. Grand mean is shown; (C) Viability of the PDAC cancer cell lines during 72 h treatment with increasing gemcitabine concentrations. Data are presented as mean ± SD normalized to T0 (n = 3; * $p < 0.05$; ** $p < 0.01$, *** $p < 0.001$); (D–E) Intracellular expression of selected miRNAs in BxPC-3 and (E) in PANC-1 cells after 72 h treatment with 20 nM gemcitabine (GEM) compared to the untreated cells. (F-G) Levels of selected sEV-miRNAs in the medium of BxPC-3 and PANC-1 cells after 72 h treatment with 20 nM gemcitabine (GEM) compared to untreated cells. Data are mean + SEM (** $p < 0.01$); Data are mean + SEM (* $p < 0.05$; ** $p < 0.01$; *** $p < 0.001$).

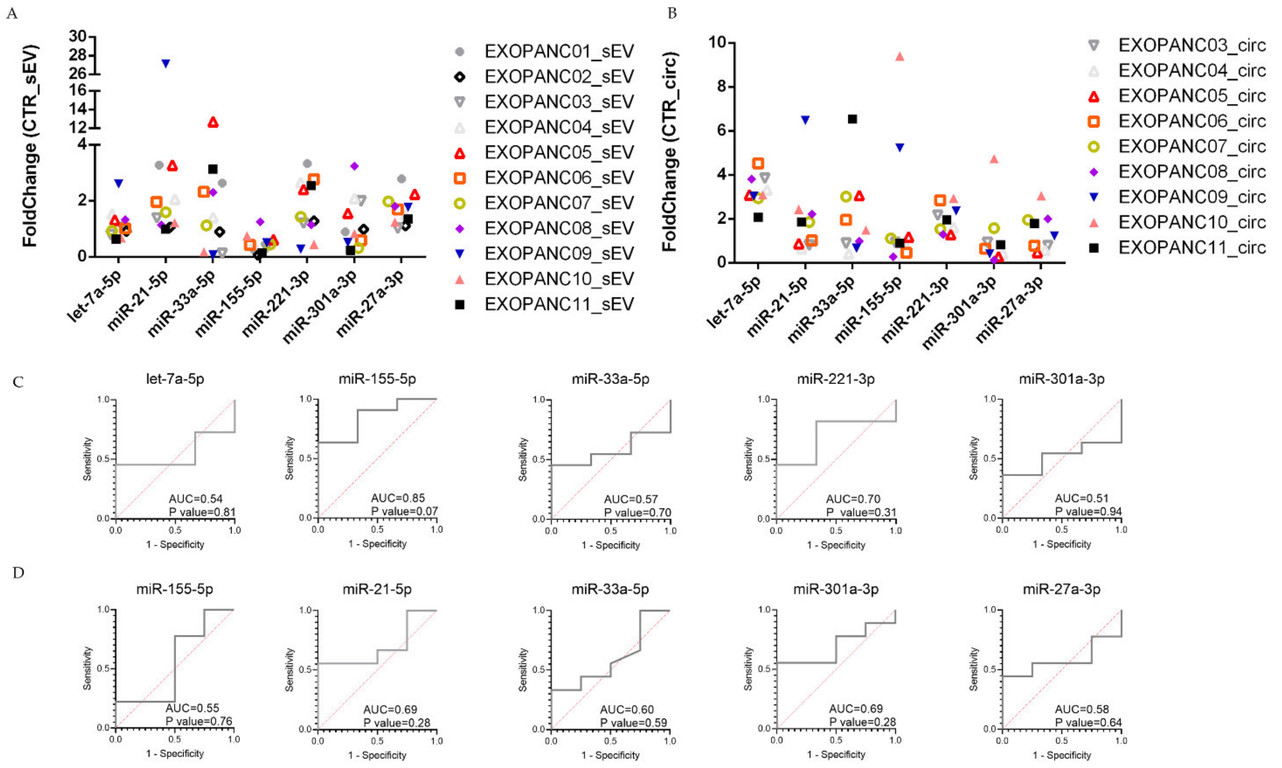

Figure S2. (A) Levels of selected vesicle-associated miRNAs in each pancreatic cancer patient (EXOPANC01-11). Fold-change between each EXOPANC patient compared to controls was reported; (B) Levels of selected circulating miRNAs in each pancreatic cancer patient (EXOPANC03-11). Fold-change between each EXOPANC patient compared to the controls was reported; (C-D) ROC curves with non-significant AUC. (C) Respective AUCs and *p* values of sEV-associated miRNAs detected in EXOPANC patients and controls quantified by RT-qPCR; (D) ROC curves of circulating miRNAs quantified by RT-qPCR extracted in EXOPANC patients and controls. The red dotted line represents an AUC threshold of 0.50. Sensitivity and 1-Specificity are represented for all of the obtained deltaCt values.

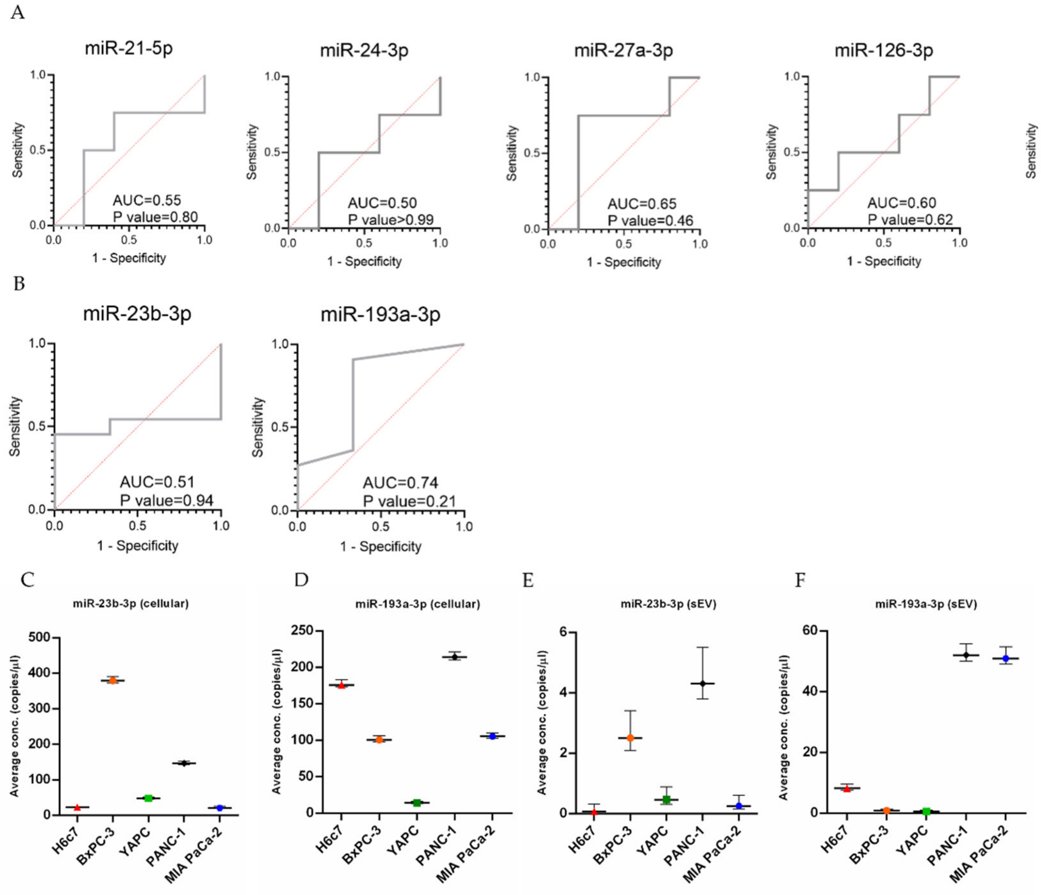

Figure S3. ROC curves with non-significant AUC. Respective AUCs and *p* values of miRNAs quantified by ddPCR. Sensitivity and 1-Specificity are represented for all the obtained copies/μL values. (A) ROC curves of circulating plasma miRNAs detected in EXOPANC patients and controls. (B) ROC curves of sEV-associated miRNAs extracted in EXOPANC patients and controls. The red dotted line represents an AUC threshold of 0.50. (C) Expression levels of miR-23b-3p and (D) miR-193a-3p in human normal and pancreatic cancer cell lines; (F) Levels of miR-23b-3p and (G) miR-193a-3p and in sEVs released in the media of pancreatic normal and cancer cells.

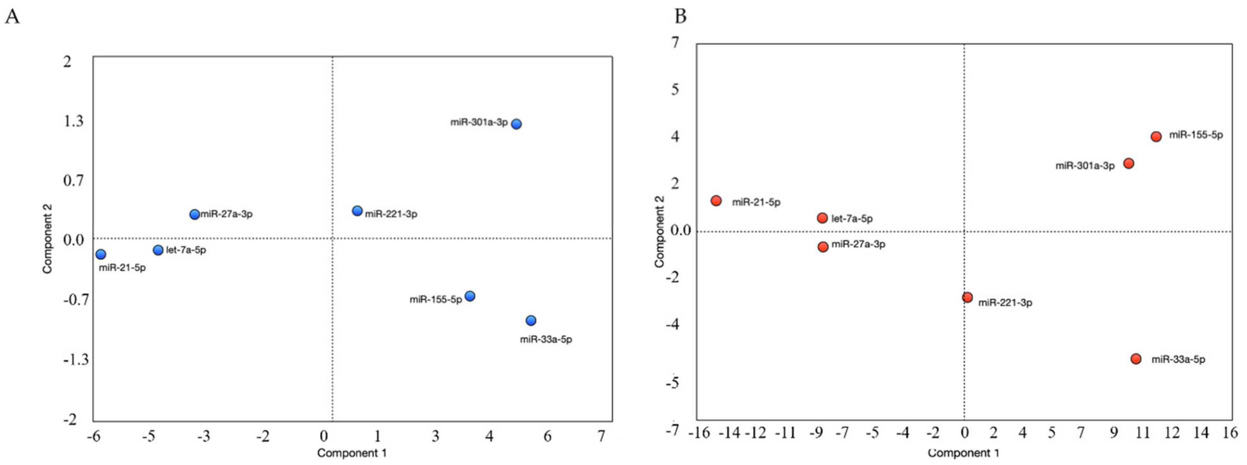

Figure S4. PCA analysis on vesicle-associated miRNAs in (A) healthy controls (blue dots) and (B) EXOPANC patients (red dots).

Table S1. Mienturnet Enrichment results as obtained from miRTarBase.

Mienturnet Enrichment results miRTarBase						
**microRNA**	**p-value**	**FDR**	**Odd ratio**	**Number of interactions**	**Target Gene 1**	**Target Gene 2**
hsa-miR-129-5p	0.051	0.202	0.187	2	*ABCB1*	*RUNDC3B*
hsa-miR-21-5p	0.0491	0.202	0.183	2	*DMTF1*	*ABCB1*
hsa-miR-221-3p	0.0191	0.202	0.11	2	*RUNDC3B*	*CROT*
hsa-miR-33a-5p	0.00478	0.202	0.0535	2	*CROT*	*RUNDC3B*
hsa-miR-1-3p	0.101	0.202	0.275	2	*SRI*	*ABCB1*
hsa-miR-155-5p	0.0978	0.202	0.27	2	*SLC25A40*	*DMTF1*
hsa-miR-16-5p	0.237	0.308	0.465	2	*DMTF1*	*RUNDC3B*

'p-value' indicates the p-values of the enrichment analysis, while 'FDR' indicates the adjusted p-values obtained by using the Benjamini-Hochberg (False Discovery Rate, FDR) procedure for multiple testing.

Table S2. Relationships between miRNAs and target genes using the miRTargetLink 2.0 platform https://ccb-compute.cs.uni-saarland.de/mirtargetlink2/ (accessed on 14 March 2024).

DMTF1	TMEM243	CROT	ABCB1	RUNDC3B	SLC25A40	DBF4	ADAM22	SRI
hsa-miR-15a-5p	hsa-miR-484	hsa-miR-17-5p	hsa-miR-1-3p	hsa-miR-10a-5p	hsa-miR-155-5p	hsa-miR-24-3p	hsa-miR-33b-3p	hsa-miR-1-3p
hsa-miR-15b-5p		hsa-miR-20a-5p	hsa-miR-9-3p	hsa-miR-10b-5p	hsa-miR-192-5p	hsa-miR-30a-5p	hsa-miR-371a-3p	hsa-let-7a-3p
hsa-miR-16-5p		hsa-miR-20b-5p	hsa-miR-21-5p	hsa-miR-16-5p	hsa-miR-215-5p	hsa-miR-30b-5p	hsa-miR-371b-3p	hsa-let-7b-3p
hsa-miR-21-5p		hsa-miR-33a-5p	hsa-miR-34b-3p	hsa-miR-29b-3p	hsa-miR-328-3p	hsa-miR-30c-5p	hsa-miR-451b	hsa-let-7f1-3p
hsa-miR-103a-3p		hsa-miR-93-5p	hsa-miR-129-5p	hsa-miR-33a-5p	hsa-miR-944	hsa-miR-30d-5p	hsa-miR-515-3p	hsa-let7f-2-3p
hsa-miR-103b		hsa-miR-106b-5p	hsa-miR-186-5p	hsa-miR-93-3p	hsa-miR-3161	hsa-miR-30e-5p	hsa-miR-519e-3p	hsa-miR-98-3p
hsa-miR-147a		hsa-miR-221-3p	hsa-miR-223-3p	hsa-miR-99a-5p		hsa-miR-98-5p	hsa-miR-539-5p	hsa-miR-1185-1-3p
hsa-miR-155-5p		hsa-miR-519d-3p	hsa-miR-451a	hsa-miR-129-5p		hsa-miR-1304-3p	hsa-miR-570-3p	hsa-miR-1185-2-3p
hsa-miR-195-5p			hsa-miR-491-3p	hsa-miR-150-5p		hsa-miR-4284	hsa-miR-624-5p	hsa-miR-3171
hsa-miR-218-5p			hsa-miR-495-3p	hsa-miR-186-3p		hsa-miR-4325	hsa-miR-770-5p	hsa-miR-4789-5p
hsa-miR-424-5p			hsa-miR-508-5p	hsa-miR-221-3p		hsa-miR-4772-3p	hsa-miR-3148	hsa-miR-5580-3p
hsa-miR-497-5p			hsa-miR-873-5p	hsa-miR-335-5p		hsa-miR-4793-3p	hsa-miR-3692-5p	
hsa-miR-503-5p				hsa-miR-483-3p		hsa-miR-6890-3p	hsa-miR-4704-5p	

hsa-miR-644a				hsa-miR-510-3p		hsa-miR-8057	hsa-miR-4712-5p	
hsa-miR-646				hsa-miR-532-3p			hsa-miR-4716-5p	
hsa-miR-3911				hsa-miR-561-3p			hsa-miR-5580-5p	
hsa-miR-4474-3p				hsa-miR-587			hsa-miR-5581-3p	
hsa-miR-4524a-5p				hsa-miR-629-3p			hsa-miR-5701	
hsa-miR-4524b-5p				hsa-miR-1301-3p			hsa-miR-6080	
hsa-miR-4720-3p				hsa-miR-2116-3p			hsa-miR-6124	
hsa-miR-6838-5p				hsa-miR-3124-3p			hsa-miR-6782-3p	
hsa-miR-6867-5p				hsa-miR-4252				
				hsa-miR-4287				
				hsa-miR-4660				
				hsa-miR-4685-3p				
				hsa-miR-4713-5p				
				hsa-miR-4766-5p				
				hsa-miR-5009-3p				
				hsa-miR-5047				
				hsa-miR-5088-3p				
				hsa-miR-6867-3p				

In orange the miRNAs identified using miRTargetLink 2.0.

Table S3. Characteristics of healthy donors.

Characteristics of healthy donors (n=8)	
Gender (F/M)	3/5
Female	37.5%
Male	62.5%
Age. mean years (range)	47.9 (40−58)

Abbreviations: F/M, Female/Male.
